# A Bovine Model of Respiratory *Chlamydia psittaci* Infection: Challenge Dose Titration

**DOI:** 10.1371/journal.pone.0030125

**Published:** 2012-01-27

**Authors:** Petra Reinhold, Carola Ostermann, Elisabeth Liebler-Tenorio, Angela Berndt, Anette Vogel, Jacqueline Lambertz, Michael Rothe, Anke Rüttger, Evelyn Schubert, Konrad Sachse

**Affiliations:** 1 Institute of Molecular Pathogenesis at ‘Friedrich-Loeffler-Institut’ (Federal Research Institute for Animal Health), Jena, Germany; 2 OIE Reference Laboratory for Chlamydiosis at ‘Friedrich-Loeffler-Institut’ (Federal Research Institute for Animal Health), Jena, Germany; 3 LIPIDOMIX GmbH, Berlin, Germany; University of California, San Francisco, University of California, Berkeley, and the Children's Hospital Oakland Research Institute, United States of America

## Abstract

This study aimed to establish and evaluate a bovine respiratory model of experimentally induced acute *C. psittaci* infection. Calves are natural hosts and pathogenesis may resemble the situation in humans. Intrabronchial inoculation of *C. psittaci* strain DC15 was performed in calves aged 2–3 months *via* bronchoscope at four different challenge doses from 10^6^ to 10^9^ inclusion-forming units (ifu) per animal. Control groups received either UV-inactivated *C. psittaci* or cell culture medium. While 10^6^ ifu/calf resulted in a mild respiratory infection only, the doses of 10^7^ and 10^8^ induced fever, tachypnea, dry cough, and tachycardia that became apparent 2–3 days post inoculation (dpi) and lasted for about one week. In calves exposed to 10^9^ ifu *C. psittaci*, the respiratory disease was accompanied by severe systemic illness (apathy, tremor, markedly reduced appetite). At the time point of most pronounced clinical signs (3 dpi) the extent of lung lesions was below 10% of pulmonary tissue in calves inoculated with 10^6^ and 10^7^ ifu, about 15% in calves inoculated with 10^8^ and more than 30% in calves inoculated with 10^9^ ifu *C. psittaci*. Beside clinical signs and pathologic lesions, the bacterial load of lung tissue and markers of pulmonary inflammation (i.e., cell counts, concentration of proteins and eicosanoids in broncho-alveolar lavage fluid) were positively associated with ifu of viable *C. psittaci*. While any effect of endotoxin has been ruled out, all effects could be attributed to infection by the replicating bacteria. In conclusion, the calf represents a suitable model of respiratory chlamydial infection. Dose titration revealed that both clinically latent and clinically manifest infection can be reproduced experimentally by either 10^6^ or 10^8^ ifu/calf of *C. psittaci* DC15 while doses above 10^8^ ifu *C. psittaci* cannot be recommended for further studies for ethical reasons. This defined model of different clinical expressions of chlamydial infection allows studying host-pathogen interactions.

## Introduction

The obligate intracellular bacterium *Chlamydia* (*C.*) *psittaci* is the causative agent of psittacosis, a widespread infection in psittacine birds and domestic poultry [1]–[3]. Transmission of *C. psittaci* to humans and the zoonotic potential of this infection were first documented in the 19th century. Outbreaks of human *C. psittaci* infections still occur [4], [5], but the number of reported cases today is thought to be underestimated due to inadequate epidemiological coverage and insufficient diagnostic testing [6], [7]. During the last decade, *C. psittaci* has also been regularly detected in non-avian domestic animals, i.e. swine, horses, dogs, cattle, and sheep [8]–[13]. Although serological data from the 1990s already indicated chlamydioses in domestic animals as a relevant source of infection for humans [14], *C. psittaci* strains of non-avian origin have not been in the focus of extensive research. Both their pathogenic role in large animals and their zoonotic potential to humans have remained elusive to date.

In natural hosts, clinical outcomes of *C. psittaci* infection range from clinical silence to severe or even life-threatening illness, suggesting that host-pathogen interactions are important to the pathogenesis. Psittacosis in birds is known as a systemic disease of acute, protracted, chronic or subclinical course. Psittacosis in humans is recognized mainly as a respiratory infection initially reminiscent of an influenza-like illness and atypical pneumonia, but may also manifest as a fulminant course including myocarditis, hepatitis, and encephalitis [15]–[17]. Diversity of chlamydial infection expression in calves ranges from acute respiratory illness, keratoconjunctivitis or polyarthritis [18]–[20] to clinically inapparent infections in the majority of herds [13]. Despite clinical silence, chlamydial infections in young cattle were found to be associated with long-lasting respiratory dysfunctions [21] indicating pathogenetic involvement of the respiratory system in “asymptomatic” bovine chlamydiosis.

Relevant animal models of chlamydial infections are needed to answer open questions about (i) the pathogenetic role of non-avian *C. psittaci* in the mammalian lung with respect to different clinical outcomes, and (ii) transmission routes of this potentially zoonotic agent between different hosts. As calves represent natural hosts for chlamydiae [20], [22], [23] they offer the possibility to analyze host-pathogen interactions under natural conditions. In contrast, artificial murine models imperfectly recapitulate many aspects of infectious diseases due to host restriction in non-typical hosts [24]. Furthermore, the following peculiarities in genetics, immunobiology and respiratory physiology reveal species-specific aspects that suggest large-animal models becoming an obligatory complement to widely used murine models.

### Genetics

The bovine genome, fully sequenced in 2009, more closely resembles the human genome than that of mice and rats [25]. Comparative analyses further revealed that sequences of bovine proteins are generally more similar to human orthologs than are rodent orthologs [26]. In general, recent data about genome diversity confirmed that the mouse genome is much more rearranged than that of most other taxa [27].

### Immunobiology

With respect to the genetically determined regulation of defense mechanisms, significant differences exist between species (reviewed by [28]). For example, interleukin-8 (IL-8) plays a significant role in human inflammatory processes. In the mouse genome, the *il-8* gene is missing; but it does exist in the genome of dogs, pigs, sheep, and cattle. The protein encoded in cattle even exhibits a high cross-species activity with human IL-8 [29], [30]. Further significant differences between murine and human innate and adaptive immune response are related to such important aspects as the Toll receptors, inducible NO synthase, Fc-Receptors, immune globulin subsets or immune mediators (summarized by [31], [32]). Particularly for chlamydial infections, marked host-adapted differences in the IFN-gamma response have been recently discovered comparing mice and humans [24].

### Respiratory Physiology

Considering the murine lung as a model for human respiratory diseases, one has to be aware of numerous structural and functional peculiarities (summarized by [33], [34]). The most important differences include the branching pattern of the bronchi (monopoidal pattern in mice *versus* dichotomous pattern in larger mammalian lungs) and the lack of bronchial vessels in mice. Due to the latter, several steps of leukocyte infiltration in the bronchial wall will be completely different compared to larger mammalian lungs. Furthermore, Clara cells are present in about 50% of airways in mice but are rare in conducting airways of humans and other larger species where goblet (mucus) cells dominate. This difference significantly influences production of mucus and consequently the function of mucociliary clearance as an important defense mechanism to eliminate inhaled particulate antigens.

That mice do not faithfully reproduce pathophysiological aspects of human pulmonary disease (due to many significant differences in lung anatomy, respiratory physiology, and pulmonary immunology) has been shown for airway epithelium repair and regeneration, asthma, cystic fibrosis, various cancers, and various pulmonary infections - for example tuberculosis or MRSA [35]–[43]. In contrast, lung volumes, airflows and respiratory mechanics are comparable between adult humans and calves due to comparable body weights (50–100 kg), and the bovine lung is particularly suited to mirror pulmonary dsyfunctions [39].

The current study was undertaken to establish and evaluate a bovine respiratory model of experimentally induced *C. psittaci* infection because calves are likely to resemble more closely than mice the situation in humans and also because chlamydial infections play an important role in cattle. As data on dose-response-relationships of chlamydial infections in the bovine respiratory system were absent, dose titration of the inoculum was the main goal of this study. Clinical outcomes, markers of pulmonary inflammation, lung pathology, recovery of chlamydiae and humoral response were assessed after intrabronchial challenge of doses between 10^6^–10^9^ inclusion forming units (ifu) per animal. Results of this study reveal that both clinically latent and clinically manifest *C. psittaci* infection can be reproduced experimentally. This defined model of a predictable severity of illness is essential for further research to understand the underlying pathogenetic mechanisms of different clinical phenotypes of chlamydial infection, and to clarify details about dissemination, shedding and transmission of *C. psittaci* as it relates to the clinical picture.

## Results

### 1. Clinical signs

**Figure 1:**
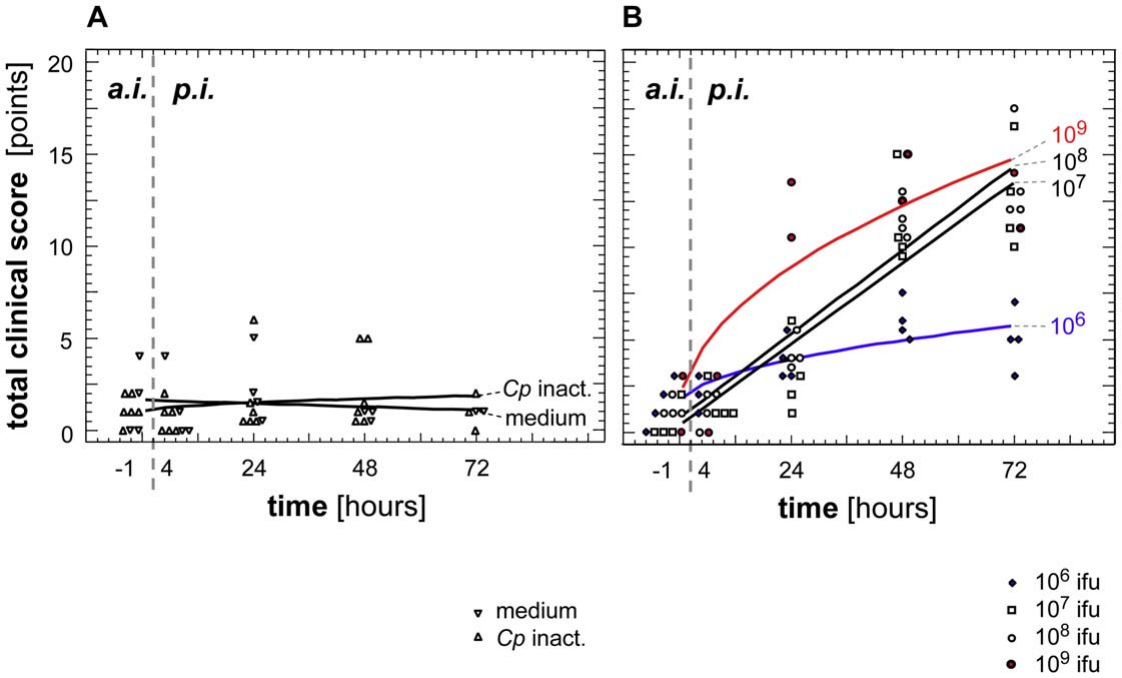
Development of the total clinical score over time. Data are given as regression lines and individual data according to the best fitting regression model per group. In control calves, no significant changes of total clinical score occurred after inoculation of medium or inactivated chlamydiae (panel A). In calves experimentally inoculated with different doses of viable *C. psittaci*, scores of clinical illness increased with challenge doses (panel B). Equations of regression, coefficients of correlation, R-squared values, and probability levels are given in Table 1.

**Figure 2:**
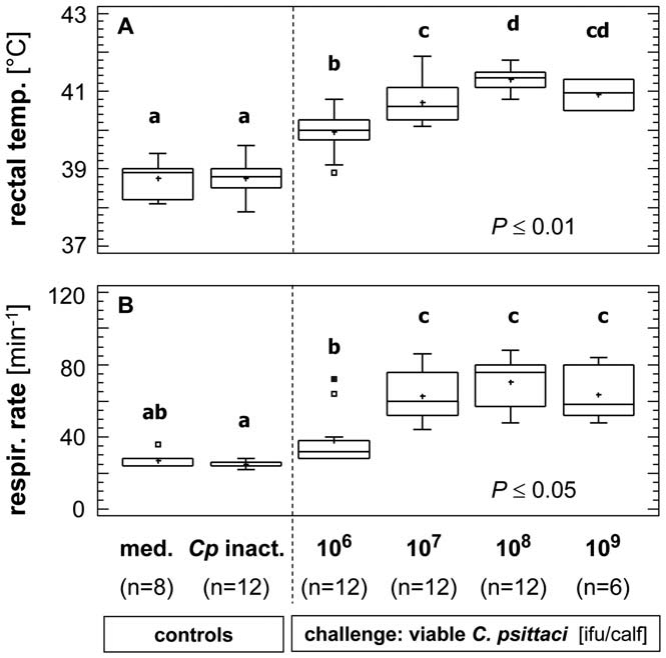
Rectal temperature and respiratory rate measured 48–72 hours post inoculation (i.e. peak of clinical signs). In calves experimentally inoculated with different doses of viable *C. psittaci,* both rectal temperature (panel A) and respiratory rate (panel B) were significantly increased while no significant changes were seen in control calves. Data are given as Box-and Whisker Plots based on 2 or 3 measurements per calf in controls or infected animals, respectively. Different letters indicate significant differences between groups at given *P*-level (multiple range test).

Control calves challenged with either cell culture medium (n = 4) or the inactivated *C. psittaci* strain (n = 6) did not exhibit any clinical sign of respiratory illness (Fig. 1A). In calves exposed to viable *C. psittaci*, the total clinical score increased with increasing doses of inoculum (Fig. 1B). Clinical illness manifested as respiratory signs and was confirmed by the respiratory score that contributed to about 50% to the general clinical score (data not shown). Clinical illness was most evident 2–3 days post inoculation (dpi). As examples, body temperatures and respiratory rates measured at the peak of clinical signs (i.e. 48–72 hours pi) are shown in Figure 2. Beside fever and respiratory illness, the following dose-dependent clinical signs were evident:

- In calves challenged with **10^6^** ifu (n = 4), mild diarrhea and spontaneous cough occurred without apparent affect on appetite, feed intake or general behavior. Nasal or ocular discharge was not observed.
- Calves exposed to **10^7^** ifu (n = 4) or **10^8^** ifu (n = 4) developed clinical illness of similar severity characterized by fever (Fig. 2A), tachypnea (Fig. 2B) and mild tachycardia (medians [ranges] of heart rates: 90 [68–120] beats min^−1^ for 10^7^ ifu/calf; 82 [72–108] beats min^−1^ for 10^8^ ifu/calf). In most of these calves, appetite and milk intake was reduced at 2–3 dpi, and diarrhea was seen in a few animals. In all calves, dry cough occurred while nasal and ocular discharges were rarely seen. In general, the period of 2–3 dpi was accompanied by reduced general activity (dullness).
- The most severe clinical picture was present in two calves challenged with **10^9^** ifu. Within the period 2–3 dpi, general behavior was mostly depressed and accompanied for 6–12 hours by apathy, inability to stand up, tremor, markedly reduced appetite or complete feed rejection with or without diarrhea. Heart rate increased to approximately 160% compared to baseline data (median [range]: 106 [88–124] beats min^−1^). Dry cough was present while nasal and ocular discharges were rarely seen. Due to severity of clinical illness, the two calves were euthanized 3 dpi and no further calves were exposed to 10^9^ ifu.

In the three groups challenged with 10^6^, 10^7^, or 10^8^ ifu of *C. psittaci*, clinical signs returned to baseline within one week after challenge (data not shown).

### 2. Biomarkers of pulmonary inflammation in BALF

Results of BALF cytology obtained during the acute phase, i.e. 2–3 dpi, are summarized in Table 2. Total cell count in BALF was higher in calves challenged with viable chlamydiae compared to controls, and counts increased with challenge dose. Although absolute numbers of all cell types (i.e. alveolar macrophages, granulocytes, and lymphocytes) contributed to elevated total cell counts in calves challenged with viable chlamydiae, the most significant increase was attributed to neutrophil granulocytes, particularly unsegmented ones. Percentages of the three cell types revealed that the relative amount of alveolar macrophages decreased significantly in a dose-dependent manner because the percentage of mainly unsegmented neutrophil granulocytes increased.

**Table 1:** Assessment of regression lines of the clinical scores given in Figure 1.

Challenge	Best fitting regression model	Coefficient of correlation	R-squared	
medium	linear [Y = a+b*X]	−0.13	1.57%	*P*>0.10
*Cp* inactivated	square root-X model [Y = a+b*sqrt(X)]	0.20	4.08%	*P*>0.10
10^6^ ifu/calf	square root-X model [Y = a+b*sqrt(X)]	0.78	61.62%	***P*** **<0.001**
10^7^ ifu/calf	Linear model [Y = a+b*X]	0.91	83.36%	***P*** **<0.001**
10^8^ ifu/calf	Linear model [Y = a+b*X]	0.96	91.40%	***P*** **<0.001**
10^9^ ifu/calf	square root-X model [Y = a+b*sqrt(X)]	0.88	77.54%	***P*** **<0.001**

**Table 2:** Quantity of cells present in broncho-alveolar lavage fluid (BALF) 2–3 days post inoculation in calves experimentally inoculated with different doses of viable *C. psittaci* (*Cp*) and in control calves.

		Control Groups		Challenge Groups				Kruskal-Wallis test
		Medium	*Cp* inactivated	*Cp* 10^6^ ifu	*Cp* 10^7^ ifu	*Cp* 10^8^ ifu	*Cp* 10^9^ ifu	
		(n = 4)	(n = 6)	(n = 2)	(n = 2)	(n = 2)	(n = 2)	
BALF cells	Unit	Med [min;max]	Med [min;max]	Med [min;max]	Med [min;max]	Med [min;max]	Med [min;max]	
Total Cell Count	10^8^/L	4.23 [1.08; 4.73]	3.38 [2.73; 4.43]	**4.83↑** [4.58; 5.08]	**6.28↑** [5.25; 7.30]	**8.72↑** [7.68; 9.75]	**11.69↑** [9.08; 14.30]	***P*** ** = 0.02**
Cell differentiation absolute								
alveolar macrophages	10^8^/L	2.94 [0.96; 4.44]	2.33 [2.10; 4.20]	3.45 [3.10; 3.80]	3.84 [3.31; 4.48]	4.51 [3.76; 5.27]	**5.00↑** [4.72; 5.29]	*P* = 0.19
neutrophil granulocytes	10^8^/L	0.27 [0.03; 1.81]	0.49 [0.04; 0.95]	1.16 [0.55; 1.78]	**2.15↑** [1.68; 2.63]	**3.89↑** [3.30; 4.49]	**5.43↑** [2.00; 8.87]	***P*** ** = 0.04**
*unsegemented*	10^8^/L	0.07 [0.00; 0.13]	0.17 [0.04; 0.25]	**0.71↑** [0.50; 0.91]	**1.01↑** [0.63; 1.39]	**2.96↑** [2.30; 3.61]	**4.63↑** [1.82; 7.44]	***P*** ** = 0.01**
*polymorph nuclear*	10^8^/L	0.20 [0.01; 1.69]	0.31 [0.00; 0.89]	0.45 [0.05; 0.86]	**1.15↑** [1.05; 1.24]	0.94 [0.88; 1.00]	0.81 [0.18; 1.43]	*P* = 0.42
lymphocytes	10^8^/L	0.14 [0.00; 0.32]	0.13 [0.08; 0.21]	0.22 [0.20; 0.23]	**0.28↑** [0.26; 0.29]	0.31 [0.00; 0.61]	1.25 [0.14; 2.36]	*P* = 0.45
Cell differentiation relative								
alveolar macrophages	%	89.5 [47; 94]	79.5 [66; 95]	72.0 [61; 83]	**61.5↓** [60;63]	**51.5↓** [49;54]	**44.5↓** [37;52]	*P* = 0.11
neutrophil granulocytes	%	6.5 [2;45]	16.5 [1;30]	23.5 [12;35]	**34.0↑** [32;36]	**44.5↑** [43;46]	42.0 [22;62]	*P* = 0.17
*unsegemented*	%	2.5 [0; 3]	5.0 [1;7]	**14.5↑** [11;18]	**15.5↑** [12;19]	**33.5↑** [30;37]	**36.0↑** [20;52]	***P*** ** = 0.01**
*polymorph nuclear*	%	4.5 [1;42]	10.0 [0; 28]	9.0 [1;17]	18.5 [17;20]	11.0 [9;13]	6.0 [2;10]	*P* = 0.71
lymphocytes	%	6.0 [0; 8]	4.0 [3;5]	4.5 [4;5]	4.5 [4;5]	4.0 [0; 8]	13.5 [1;26]	*P* = 0.99

Med = median. Kruskal-Wallis test: *P*≤0.05 indicates significant differences between medians of all groups. *W* test: *P*≤0.067 indicates significant differences between two groups. Medians highlighted in bold increased (**↑**) or decreased (**↓**) significantly in comparison to controls ‘*Cp* inactivated’. Data of the two control groups did not differ significantly.

BALF cytology of the calves that had been exposed to viable *C. psittaci* and necropsied at 7 dpi still showed dose-dependent effects. For example, total cell counts 7 days after exposure to 10^6^, 10^7^, and 10^8^ ifu were still 4.8, 7.0, and 8.2×10^8^/L, respectively. In calves sacrificed at 14 dpi, BALF cytology did not differ from those of control calves (data not shown).

**Figure 3:**
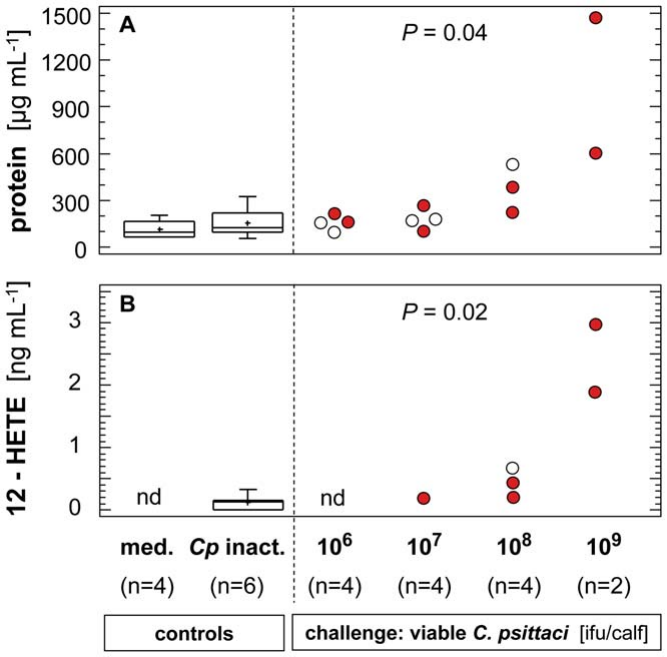
Markers of pulmonary inflammation assessed in broncho-alveolar lavage fluid (BALF). Both concentration of total protein (A) and 12-HETE (B) were maximal in calves inoculated with 10^9^ ifu of *C. psittaci*. Data for control groups are from 2 and 3 dpi combined (Box and Whisker Plots). Data for calves challenged with different doses of viable *C. psittaci* are given on an individual basis for time points when calves were sacrificed (filled circles: 3 dpi; open circles: 7 dpi and 14 dpi). Kruskal-Wallis test revealed significant differences between groups at given *P*-level.

The concentration of total protein in BALF supernatant was <300 µg/mL in controls as well as in groups challenged with 10^6^ or 10^7^ ifu. Protein concentration in BALF increased in the group challenged with 10^8^ ifu, and was dramatically elevated after inoculation of 10^9^ ifu of *C. psittaci* (Fig. 3A).

As shown in Figure 3B as a typical example, eicosanoids, i.e. thromboxan B2 (TXB2), prostaglandin E2 (PGE2), and hydroxyeicosatetraenoic acids (15-HETE, 12-HETE), were almost undetectable in BALF supernatants of the control animals but became measurable in calves challenged with doses above 10^6^ ifu and attained highest concentrations in BALF samples of calves exposed to 10^9^ ifu.

### 3. Pulmonary lesions and detection of chlamydiae

#### 3.1. Gross lesions

**Figure 4:**
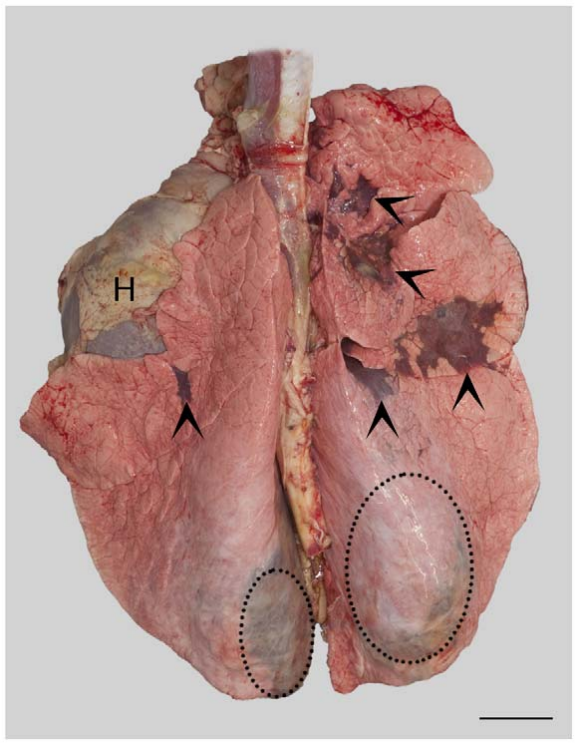
Distribution and extent of pulmonary lesions at day 3 pi in a calf inoculated with 10^9^ ifu of *C. psittaci*. Dorsal view of the lung and heart (H). Pneumonic lesions present as dark red discolorations (>) in the apical lobes, middle lobe and basal lobes. Note distension of the basal lobes due to severe bronchopneumonia in the inferior segments (hatched lines). Bar = 5 cm.

Bronchopneumonia was seen in all calves exposed to viable *C. psittaci*, but in none of the calves inoculated with cell culture medium or inactivated *C. psittaci*. Distribution of lesions was consistent with the sites where inoculum had been applied. Thus, the most extensive involvement was seen in the middle lobe and in the left and right basal lobes (Fig. 4). Especially in the basal lobes, lesions were often not readily visible at the surface, but located deep within the tissue (Fig. 4). At the time point of most pronounced clinical signs, i.e. 3 dpi, the extent of lesions was below 10% of pulmonary tissue in calves inoculated with 10^6^ and 10^7^ ifu *C. psittaci*. The proportion increased to about 15% in calves inoculated with 10^8^ and to more than 30% in calves inoculated with 10^9^ ifu *C. psittaci*.

In calves inoculated with 10^6^, 10^7^ and 10^8^ ifu *C. psittaci* necropsied at 7 dpi, lesions were still present in the basal lobes, but at 14 dpi lesions had almost resolved.

#### 3.2. Histological lesions and detection of chlamydial inclusions by immunohistochemistry

Neither histological lesions nor chlamydial inclusions were seen in the calves inoculated with medium or with inactivated chlamydiae. In the calves challenged with viable chlamydiae, the presence of *C. psittaci* inclusions was mainly restricted to altered pulmonary tissue, and macroscopic lesions were confirmed by histology as follows:

At 3 dpi, purulent bronchopneumonia was seen predominantly in calves inoculated with 10^6^ and 10^7^ ifu *C. psittaci*. Small foci with fibrinous exsudate and necrosis were seen only in severely affected lobes. The number of chlamydial inclusions was low, and had a multifocal distribution. Chlamydial inclusions were seen in alveolar epithelial cells. After inoculation of 10^8^ ifu *C. psittaci,* fibrinopurulent bronchopneumonia with multifocal areas of necrosis and pleuritis was frequently observed. The number of chlamydial inclusions was further elevated and the inclusions were often associated with neutrophils and macrophages. In calves inoculated with 10^9^ ifu *C. psittaci*, areas of necrosis were more extensive and numerous chlamydial inclusions were found throughout the altered tissues.

At 7 dpi, an increased number of alveolar macrophages and mild lymphohistiocytic infiltrates occurred, indicating organization of pneumonic lung tissues. Extensive areas of necrosis were seen in the calf inoculated with 10^8^ ifu *C. psittaci,* multifocal areas in the calf inoculated with 10^7^ ifu *C. psittaci* and none in the calf inoculated with 10^6^ ifu *C. psittaci.* Chlamydial inclusions were numerous in areas of necrosis, but infrequent in those of organization.

At 14 dpi, lesions had resolved in the calf inoculated with 10^6^ ifu *C. psittaci.* The lung of the calf that had received 10^7^ ifu *C. psittaci* had multiple areas with thickened interalveolar septae, alveolar epithelial cell type II hyperplasia and lymphocytic infiltrates. Few chlamydial inclusions were found overall, but there were a few foci with groups of macrophages containing chlamydial inclusions.

### 4. Quantification of chlamydial antigen in the lung

**Figure 5:**
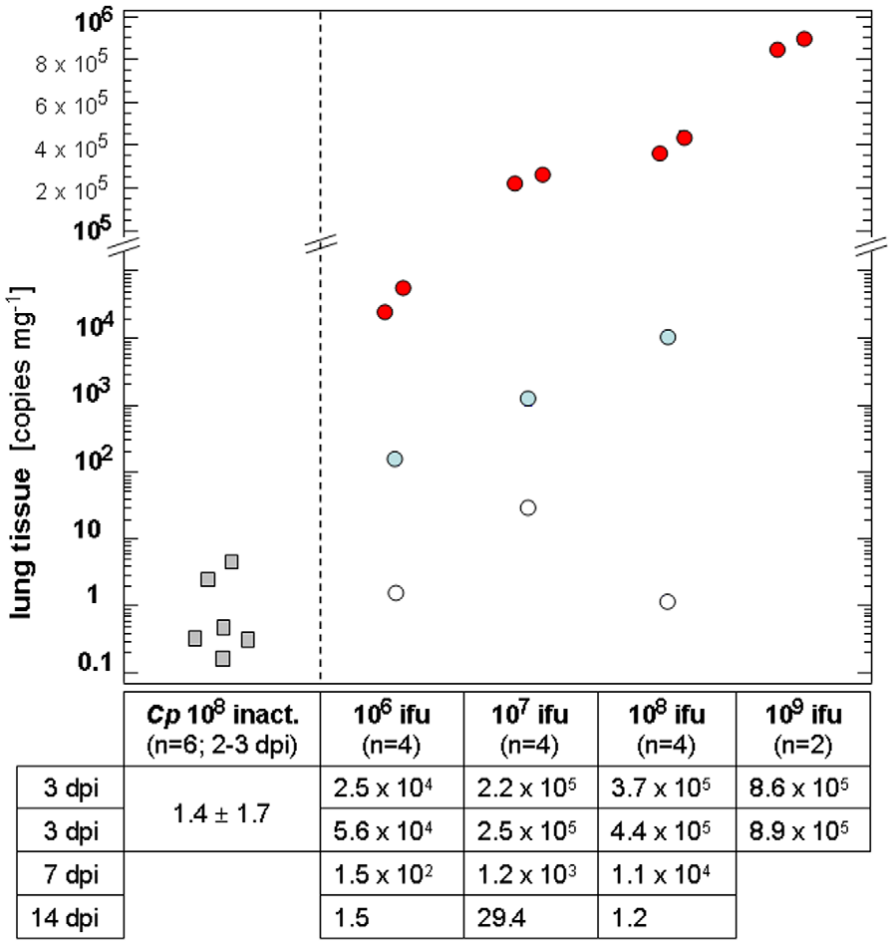
Numbers of inclusion-forming units (ifu) of *C. psittaci* in lung tissues. Data are expressed as individual animals. Boxes indicate control calves euthanized 2–3 dpi after inoculation of UV-inactivated 10^8^ ifu. Circles represent calves challenged with viable bacteria of different doses. Infected calves were sacrificed 3 dpi (red circles), 7 dpi (blue circles) and 14 dpi (open circles). Copy numbers in lung tissue represent the mean of 4 samples analyzed per lung (two of left caudal lobe and two of right caudal lobe).

Examination of lung tissue by real-time PCR revealed that genome copy numbers of *C. psittaci* in lung tissue at 3 dpi increased with the challenge dose (Figure 5). Seven days after challenge, copy numbers of the pathogen were still dose-dependent but already significantly reduced compared to 3 dpi. Fourteen days post inoculation, less than 30 copies/mg were detectable in lung tissues of calves challenged with viable chlamydiae.

In lung lymph nodes, similar dose-dependent effects were seen at 3 dpi, but absolute copy numbers per mg of lymph node tissue were much lower compared to those found in lung tissue (data not shown). In cell pellets of BALF, highest copy numbers were seen at 3 dpi in the two calves challenged with the highest dose of 10^9^ ifu/calf (621 and 1611 copies per 10^4^ BALF-cells), while hardly any *C. psittaci* were found in BALF cells of the other groups at any time point.

### 5. Humoral response

Specific antibodies against the challenge pathogen were detected mainly in the group of animals exposed to 10^8^ ifu *C. psittaci*, where reactive bands were detected in serum on day 7 after inoculation (Fig. S1). Sera from the groups infected with 10^7^ and 10^6^ ifu failed to show a specific immune reaction in the first 11 days after challenge. Immunoblot analysis of BALF supernatants showed only a weak reaction for the group exposed to 10^8^ ifu on 7 dpi (Fig. S1). Since the two calves challenged with 10^9^ ifu had been sacrificed already at 3 dpi, no data of their specific humoral response is available.

## Discussion

### 1. Model validity

To the best of our knowledge, this is the first study assessing dose response effects to the pathogen *C. psittaci* in a domestic animal model of respiratory infection. Despite the known disadvantages in terms of cost, time consumption and limited availability of immunological and molecular tools compared to widely used murine models, calves were selected because (1) bovine chlamydiosis closely resembles the situation in a natural host [21], [23], (2) the *C. psittaci* isolate (strain DC 15) used to establish the model originated from a calf, and (3) the bovine lung is more relevant than the mouse to model human functional consequences of ventilatory disorders due to its segmental anatomy and the lack of collateral airways [39]. Furthermore, domestic animal models are especially advantageous because they can be used as dual-purpose models that benefit both agricultural and biomedical research [44], [45]. From an epidemiological point of view, infectious diseases in farm animals are useful biological models to provide empirical data that aids infectious disease modeling and to advance our understanding of infectious disease dynamics and control for human populations [46].

Due to ethical criteria of animal protection, calves included were limited to the lowest number essential to document inherent differences in host responses (4 calves per challenge dose 10^6^–10^8^ ifu/calf; 2 calves per challenge dose 10^9^ ifu/calf). This small animal number was sufficient because large animals offer the great advantage of enabling the characterization of functional, inflammatory and morphological changes in a multi-factorial within-subject approach.

With respect to the pathogen, previous models of respiratory ‘*C. psittaci* infection’ in domestic animals published more than 20 years ago have to be critically scrutinized on the basis of current taxonomy. For instance, isolates of *C. psittaci* from ovine pneumonia were inoculated either endobronchially in red deer [47] or intratracheally in pigs [48] to produce pneumonia. Experimentally induced pneumonia by intratracheal inoculation of different strains of the old *Chlamydia psittaci sensu lato* (now comprising the species of *C. abortus*, *C. felis*, *C. caviae* and *C. psittaci*) was also reported for pigs and calves [49]–[51]. From today's perspective, in the light of two recent revisions of the taxonomy of *Chlamydiales* [52], [53], it is doubtful that those models actually used the species currently defined as *C. psittaci*.

### 2. Dose-dependent effects of *C. psittaci* in the host

Clinical signs of illness increased with challenge doses in all calves exposed to viable *C. psittaci*. While 10^6^ ifu/calf resulted in mild clinical signs only, the doses of 10^7^ and 10^8^ induced clinically apparent illness that became visible 2–3 dpi. Comparing the latter two doses, the clinical picture induced by 10^8^ ifu was more reproducible. Doses above 10^8^ ifu *C. psittaci* cannot be recommended for further studies for ethical reasons.

According to BALF cytology, increasing numbers of cell types capable of phagocytosis and antigen presentation were recruited with increasing challenge doses of *C. psittaci*. The early recruitment of neutrophils is in line with results obtained after aerogeneous *C. suis* infection in pigs [54] and in a murine model of *Chlamydia* infection [55]. In our model, predominantly juvenile unsegmented neutrophils were recruited as the first line of defense in a dose-dependent manner. To a smaller extent, alveolar macrophages and lymphocytes also contributed to the increase of cells in broncho-alveolar compartments.

Concentrations of total protein in BALF indicated severity of inflammation and increased permeability of pulmonary vessels for challenge doses above 10^7^ ifu/calf. Increases in protein concentration >600 µg/mL BALF as seen after inoculation of 10^8^ ifu of *C. psittaci* are in accordance with data published for calves with naturally acquired chlamydial infections [21]. Dramatically elevated protein concentrations in BALF samples of the two calves exposed to 10^9^ ifu of *C. psittaci* demonstrated dramatic loss of integrity of the alveolo-capillary barrier in the lung, which is line with the particularly severe clinical outcome.

The pathogenetic link between chlamydial infection and activation of the arachidonic acid (AA) cascade *via* the cyclooxygenase (COX)-mediated pathway has been shown *in vitro* for multiple cell types such as epithelial cells, peripheral blood mononuclear cells, human monocytes, and antigen-presenting dendritic cells [56]–[59]. In our model, eicosanoids produced by this pathway were increasingly obvious with increasing challenge doses indicating that the AA cascade became more intensively involved in pulmonary host response as the chlamydial inoculum increased. This finding is in good agreement with *in vitro* data obtained in human monocytes showing that the amount of synthesized eicosanoids was dependent on the chlamydial multiplicity of infection [60]. While *in vitro* studies focused mainly on PGE2, *in vivo* data of our study revealed that concentrations of at least four eicosanoids (TXB2, 15-HETE, 12-HETE, and PGE2) increased with chlamydial load in lung tissue.

Differences in the quantity of pulmonary tissue affected by pneumonia were well correlated with the severity of clinical signs. A dose-dependent increase in the number of pulmonary lesions and in the type of lesions was observed. There was a continuous change from purulent to fibrino-exsudative lesions and in the extent of necrosis. Similar changes may be seen after deposition of foreign material in the lung causing aspiration pneumonia. However, since lesions occurred after inoculation with viable chlamydiae only, they are most likely a consequence of the replicating bacteria. Early organization of pulmonary lesions was seen at 7 dpi. Chlamydiae were still present in areas of inflammation, but had been cleared from areas of organization. At 14 dpi, reconstitution was complete in calves that had received 10^6^ ifu of *C. psittaci*. In calves that had received the higher doses, areas of necrosis had not yet been completely organized and chlamydiae could still be found in these areas.

Specific antibodies against *C. psittaci* occurred in both blood and BALF about 7 dpi, but only in calves exposed to the challenge dose of 10^8^ ifu. Lower challenge doses did not induce a measurable specific humoral response within two weeks after inoculation. Whether humoral response to lower challenge doses requires a longer time or whether challenge doses below 10^8^ ifu are not sufficient to induce humoral response has yet to be elucidated.

### 3. Detection of the pathogen: Localization and time-dependence

Within 14 days after intrabronchial challenge with viable *C. psittaci*, different kinds of swabs (nasal, ocular, rectal) collected on a daily basis were unsuitable to detect the challenge strain by PCR (data not shown). In lung tissue, however, *C. psittaci* was detected by real-time PCR, and increasing copy numbers of the challenge strain were found in correspondence to increasing challenge doses until 7 dpi. Furthermore, we were able to recover the challenge strain in cell culture from lung tissues obtained at necropsy (data not shown).

The presence of chlamydial inclusions assessed by immunohistochemistry was restricted to altered pulmonary tissue while alterations mainly surrounded the eight locations of inoculation. Distribution of any infection or inflammation in the bovine lung is spatially hampered due to a very high degree of lobulation and segmentation of pulmonary tissue and the lack of collateral airways [39], [61], [62]. Thus, dissemination of infection was impossible *via* connective tissue septa between pulmonary segments and could only happen if infectious particles or droplets would be transported to other segments by airflow. This however, is less likely for obligate intracellular pathogens such as chlamydiae. Consequently, consolidated lobules could be found adjacent to healthy lobules within the same lung lobe.

### 4. Exclusion of effects mediated by lipopolysaccharide or liquid instillation

Inoculation of cell culture medium did not result in any clinical sign or pulmonary lesion, excluding significant host response to instillation of 6 ml liquid per lung.

To address the question to what extent chlamydial lipopolysaccharide (LPS) induced either local effects in the lung or general clinical signs, we included a second control group that was inoculated with UV-inactivated *C. psittaci* at the highest acceptable dose (10^8^ ifu). Compared to controls exposed to cell culture medium only, calves exposed to inactivated chlamydiae did not express any significant difference in any parameter assessed in this study. Consequently, clinical signs of respiratory disease and local effects of inflammation induced in the lung required viability of the pathogen. The time course of alterations in calves challenged with doses 10^6^ to 10^8^ ifu is consistent with the duration of at least one chlamydial developmental cycle in the host cells (initial clinical signs occurred about 48 h after challenge). In calves exposed to 10^9^ ifu of *C. psittaci,* signs of general clinical illness occurred earlier (less than 24 h after challenge) which might indicate involvement of toxic products from the pathogen or stronger release of inflammatory mediators by the host. For challenge doses 10^6^ to 10^8^ ifu per calf, however, involvement of LPS effects in the pathogenesis could be excluded.

### 5. Conclusions

The calf was found to be a suitable mammalian host to establish and evaluate an *in vivo* model of experimental respiratory infection by *C. psittaci*. Intra-bronchial challenges between 10^6^ to 10^9^ ifu/calf resulted in dose-dependent pulmonary and systemic host reactions ranging clinically from mild to severe. For further studies, only doses between 10^6^ and 10^8^ ifu per animal are recommended, depending on the clinical outcome to be achieved. While 10^6^ ifu of strain DC 15 per animal will lead to a mild or even subclinical infection, 10^8^ ifu per animal causes reproducible clinically manifest disease and predictable humoral response.

This domestic animal model will add valuable information to the current knowledge about chlamydial infections obtained from other studies (laboratory animal or cell culture models). It may be used to address the following questions with relevance for both human and veterinary medicine:

1. To study pathogenetic details of *C. psittaci* infection at the tissue level, i.e. the interplay between intracellular chlamydial infection and host cell responses.
2. To verify consequences of *C. psittaci* infection at the organ level, i.e. pulmonary dysfunctions in the host.
3. To characterize long-term host-pathogen interactions *in vivo.*
4. To assess the spread and shedding of the organism in order to understand the dissemination of the pathogen within the host and transmission routes between animals, as well as from animals to humans.
5. To evaluate the usefulness and efficacy of prophylactic and therapeutic options in order to control chlamydioses in livestock and, perhaps, eliminate chlamydial infections in human patients.

## Materials and Methods

### 1. Legislation and ethical approval

This study was carried out in strict accordance with European and National Law for the Care and Use of Animals. The protocol was approved by the Committee on the Ethics of Animal Experiments and the Protection of Animals of the State of Thuringia, Germany (Permit Number: 04-002/07). All experiments were done in a containment of biosafety level 2 under supervision of the authorized institutional Agent for Animal Protection. Bronchoscopy to inoculate the pathogen was strictly performed under general anesthesia. During the entire study, every effort was made to minimize suffering.

### 2. Animals

In this prospective and double-controlled study, 24 conventionally raised calves (Holstein-Friesian, male) were included. Animals originated from one farm without any history of *Chlamydia*-associated health problems. Before the study, the herd of origin was regularly checked for the presence of chlamydiae by the National Reference Laboratory for Psittacosis. Calves were purchased at the age of 16 to 26 days weighing between 48 and 76 kg (57±6; mean ± SD). After a quarantine period of at least 20 days and confirmation of a clinically healthy status, animals were included in the study.

Throughout the entire study, animals were reared under standardized conditions (room climate: 18 to 20°C) and in accordance with international guidelines for animal welfare. Nutrition included commercial milk replacers and coarse meal. Water and hay were supplied *ad libitum*. None of the given feed contained antibiotics.

### 3. Study design

At the age of 45–54 days, 14 calves weighing 70.8±4.3 kg were inoculated with *C. psittaci* whereas another 10 calves (body weight: 71.6±7.2 kg) served as controls. By bronchoscope, four challenge groups received four different infection doses of live *C. psittaci* containing the following amounts of inclusion-forming units (ifu) in 6 mL stabilizing medium SPGA (containing **s**accharose, **p**hosphatile substances, **g**lucose and bovine **a**lbumin; [63]: 10^6^ (n = 4), 10^7^ (n = 4), 10^8^ (n = 4), and 10^9^ (n = 2) ifu per animal, respectively. Controls received either 6 mL containing 10^8^ ifu of inactivated strain DC 15 (n = 6) or cell culture medium colored by ink solution (5 mL per animal; dilution: 1∶5).

Animals exposed to 10^6^–10^8^ ifu of were euthanized and necropsied 3, 7 or 14 days post inoculation (dpi), while the two calves exposed to 10^9^ ifu were sacrificed 3 dpi. Controls were euthanized 2 and 3 dpi. Broncho-alveolar lavage was performed, and lungs were examined and sampled to assess lesions and presence of *C. psittaci*.

Before inoculation until necropsy, each calf underwent daily clinical examination. In addition, blood samples were collected daily to monitor humoral immune response. Thus, venous blood was collected from the jugular vein before morning feeding using 9.0 mL plastic syringes (S-Monovette, Sarstedt AG & CoKG, Nuembrecht, Germany). Serum was harvested by centrifugation and stored at −20°C until analyzed.

### 4. Preparation of bacteria used for inoculation

#### 4.1. Live chlamydiae

Strain DC 15 was isolated at Friedrich-Loeffler-Institut (Jena, Germany) from an aborted calf fetus in 2002. The isolate was classified as *C. psittaci* genotype A-VS1 by DNA microarray testing and *omp*A gene sequencing [64]. Chlamydiae were propagated in buffalo green monkey kidney (BGM) cell culture using standard procedures [65]. Frozen stocks of strain DC15 were diluted to the required titer in stabilizing SPGA medium and used as antigen in the present trial.

#### 4.2. Inactivated chlamydiae

Six-well cell culture plates were filled with 7-mL portions of stabilizing medium containing 10^8^ ifu of *C. psittaci* DC15. Inactivation was achieved by 4.5-h exposure on a UV Transilluminator plate (UVP Inc. CA, Upland, CA) and simultaneous irradiation from a UV lamp installed above the vessel. While 6 mL were preserved as a single-animal dose to be inoculated, the remains of about 1 mL were left for subsequent examination of viability of these preparations. Cell culture passages using immunofluorescence confirmed the inability of treated chlamydial bodies to re-enter a developmental cycle.

### 5. Intrabronchial administration

For intrabronchial challenge, the non-fed calf was anesthetized with xylazin (0.2 mg/kg bodyweight, Rompun 2%, Bayer Vital GmbH, Leverkusen, Germany) and ketamine (1.7±0.3 mg/kg bodyweight, Ursotamin, Serumwerk Bernburg AG, Bernburg, Germany); both injected intravenously at time intervals of approximately 3 min.

For inoculation, a flexible video endoscope of 140 cm working length and outer diameter of 9 mm was used (Veterinary Video Endoscope PV-SG 22–140, KARL STORZ GmbH & Co.KG, Tuttlingen, Germany). The endoscope was inserted through a metal tubular speculum (diameter: 3.5 cm, length: 35 cm) placed into the calf's mouth. Defined doses of the freshly prepared *C. psittaci-*suspension or cell culture medium, respectively, were administered at eight defined locations in the lung (Fig. S2) using a Teflon tube (inner diameter 1 mm, outer diameter 2 mm, 175 cm length, dead space: 1.4 mL) that was inserted through the working channel (diameter 2.2 mm) of the endoscope.

### 6. Clinical Scoring

Clinical observations were recorded twice daily and included feed intake, rectal temperature, respiratory rate, and the presence or absence of clinical signs of diarrhea or respiratory disease, such as cough or nasal discharge. In addition, the appearance of oral mucosa, conjunctivae, skin, hair and dyspnea were assessed daily, and the heart rate was counted. Extremities, umbilicus and *Lnn. mandibulares* were palpated and inducement of cough was tested (by a short compression of the larynx). Results were summarized using a 49-point clinical score (Table S1) consisting of sub-scores for general condition (max. 8 points), respiratory system (max. 17 points), cardiovascular system (max. 13 points) and other organ systems (max. 11 points).

### 7. Necropsy and tissue samples

At the end of the study, all animals were euthanized. Under conditions of deep anesthesia (pentobarbital-sodium, 770±123 mg/10 kg bodyweight, intravenously, Release, WdT eG, Garbsen, Germany), the trachea was exposed and large clamps were placed distal to the larynx to prevent contamination of the airways by blood or gastric contents. Subsequently, the animals were sacrificed by exsanguination. The lung was removed, macroscopic lesions recorded and samples collected from each lung lobe. Sites with macroscopic lesions were preferentially sampled. Aliquots of each sample were used for histological and immunhistological examination and detection of *C. psittaci* by PCR. Then a complete necropsy was performed.

### 8. Collection of broncho-alveolar lavage fluid and BALF analyses

Broncho-alveolar lavage fluid (BALF) was obtained from freshly exenterated lungs immediately after exsanguination. At three different locations (*Lobus caudalis dexter, Lobus medius, Lobus caudalis sinister*) three subsequent washes using 20 mL of ice-cold cell buffer (140 mM NaCl; 2.8 mM KCL; 10 mM Na_2_HPO_4_×12H_2_O) for each instillation (in total 180 mL; 60 mL per lung lobe) were installed using glass syringes and a catheter inserted through the trachea. BALF obtained by aspiration was immediately placed on ice. The BALF recovery was 55±6% (mean ± SD) and did not differ between groups. Cells and supernatant of BALF were separated by centrifugation (300× *g*; 20 min).

BALF cytology — Absolute number of leukocytes in BALF was determined by cell counting using traditional ‘Neubauer chambers’. To quantify leukocyte populations, 400 µL of native BALF were placed on glass slides. The cellular sediments were fixed with 100% methanol for 10 min and subsequently stored at −20°C. For microscopic examination, the cell sediments were stained according to Pappenheim (HemaDiff, bioanalytic GmbH, Umkirch/Freiburg, Germany), and the percentages of leukocyte populations (lymphocytes, macrophages, unsegmented and polymorphonuclear neutrophil granulocytes) were determined by counting a total of 100 cells. The absolute cell numbers of the leukocyte subsets in BALF were calculated based on the absolute number of leukocytes and the percentages of leukocyte populations.

Total protein — Concentrations of total protein were measured in BALF supernatant using commercially available modified Lowry Protein Assay Kit (Pierce, Rockford IL, USA). Each sample was analyzed in duplicate.

Eicosanoids — Liquid Chromathography – Tandem Mass Spectrometry (LC-MS-MS) was used to analyze concentrations of TXB2, PGE2, 15-HETE, and 12-HETE in BALF supernatant. Lipid mediators and the deuterated standards were purchased from Cayman Chemical (Ann Arbor, USA). Solvents and reagents (water, methanol, acetonitrile, formic acid and ammonium acetate) were LC-MS grade from Fisher Scientific (Loughborough, United Kingdom). After adding internal standards, the samples were filtrated and directly analyzed using an Agilent 1200 HPLC system coupled with an Agilent 6460 Triplequad mass spectrometer with electrospray ionisation. HPLC conditions were as follows: Zorbax Stable Bond 3.5 µm, 2.1×150 mm column, injection volume 20 µL, flow rate 0.4 mL/min, elution gradient from 10% (v/v) acetonitrile to 90% in 10 min, held for another 10 min. Analysis of lipid mediators was performed with Multiple Reaction Monitoring in negative mode. Results were calculated using the Agilent Mass Hunter Software.

### 9. Gross pathology, histopathology, immunohistochemistry

Distribution, extent and quality of macroscopic pulmonary lesions were recorded. Tissues collected at necropsy were fixed in 3.5% neutral buffered formalin for 24 h and embedded in paraffin. Lesions were evaluated in hematoxylin- and eosin-stained paraffin sections. Chlamydiae were labeled in paraffin sections by indirect immunoperoxidase method using the anti chlamydial-LPS antibody ACI-P500 (Progen, Heidelberg, Germany) as primary antibody and peroxidase-labeled sheep anti-mouse IgG (NA 931, GE Healthcare Europe GmbH, Freiburg, Germany) as secondary antibody. Sections were pre-digested with 0.05% proteinase K (Merck, Darmstadt, Germany) for antigen retrieval.

### 10. Detection and quantification of chlamydiae using real-time PCR

Samples of lung tissue (2 of left caudal lobe, 2 of right caudal lobe), lung lymph nodes, and BALF-cells were subjected to DNA extraction using the High Pure PCR Template Preparation Kit (Roche Diagnostics, Mannheim, Germany) following the instructions of the manufacturer. One µl of the final eluate was used as template in real-time PCR testing for the family *Chlamydiaceae* [66] and the species of *C. psittaci* [67].

### 11. Immunoblotting

Immunoblotting was applied to both sera and BALF to detect specific antibodies. Lysates of partially purified elementary bodies of *C. psittaci* strain DC15 were separated by sodium dodecyl sulfate polyacrylamide gel electrophoresis (SDS-PAGE) under reducing conditions using a standard protocol. BGM cell lysates were included as controls. Prior to electrophoresis, the protein content had been determined using the Bradford reagent (Sigma, Hamburg, Germany), so that equal amounts of protein, i.e. 5 µg per lane, could be run from each sample. Semi-dry electroblotting was used to transfer the separated bands onto polyvinylidene difluoride membranes (PVDF, Amersham Biosciences, NJ, USA). Subsequently, membranes were blocked with 5% (weight/volume percent; w/v) skimmed dried milk (Roth, Karlsruhe, Germany) in TBS-T (10 mM Tris-HCl, 0.15 M NaCl, 0.1% Tween-20, pH 7.4) for 1 h and probed overnight with serum or BALF supernatant (both 1∶50 dilution in TBS-T). Thereafter, incubation of Protein G conjugated to horseradish peroxidase (HRP Calbiochem, Nottingham, UK) was generally performed in 1% (w/v) bovine serum albumine (Serva, Heidelberg, Germany) in TBS-T. The blots were stained by adding the HRP substrate chloronaphthol (Sigma, Hamburg, Germany) and photographed using a G:Box imager and GeneSnap software (Syngene, Cambridge, UK).

### 12. Exclusion of co-infections

The herd of origin was known to be free of bovine herpes virus 1 (BHV-1) and bovine virus diarrhoea/mucosal disease virus (BVDV). Routine microbiological screening revealed that all animals were negative for Salmonella infections (fecal swabs) and relevant enteric parasites (fecal smearing). To verify relevant respiratory co-pathogens, the presence of *Mycoplasma*, *Pasteurella* or *Mannheimia* spp. was evaluated in nasal swabs taken immediately before challenge and before necropsy as well as in lung tissue samples obtained during necropsy. Neither *Mannheimia haemolytica* nor *Mycoplasma bovis* was detected in any sample. *Pasteurella multocida* and *Mycoplasma bovirhinis* was detected at least once in a nasal swab from 4 of 24 calves (17%) or 8 of 24 calves (33%), respectively, but never in any lung tissue sample. By serology, systemic infection with *Mycoplasma bovis* could be excluded (ELISA Kit for *Mycoplasma bovis,* Bio-X-Diagnostics, Jemelle, Belgium). Serology was also used to check for viral co-pathogens i.e. bovine respiratory syncytial virus (BRSV), parainfluenza 3 virus (PI-3), adenovirus type 3, BHV-1 and BVDV (Bio-X respiratory penta ELISA Kit, Bio-X-Diagnostics,). Only maternal antibodies (with titers decreasing in the course of the study) were seen against BRSV (24/24), PI-3 (24/24), and adenovirus type 3 (23/24). In addition to serology, ear biopsies were examined for the presence of BVDV by immunohistochemistry [68]. All biopsies were negative for BVDV antigen indicating that none of the calves was immunocompromised by persistent BVDV infection.

During the quarantine period, all 24 calves included were checked serologically for antibodies against chlamydiae (ELISA test; IDEXX GmbH, Ludwigsburg, Germany). While 23/24 were serologically negative prior to inoculation, one calf (later challenged with inactivated chlamydiae) revealed an unexpected positive test result.

### 13. Statistical analysis

Data with normal distribution are presented as mean and standard deviation (SD) while data with non-normal or unknown distribution are given as median and range (minimum-maximum). Box and Whisker Plots represent lower and upper quartile values (box) with median and mean (+). Whiskers extend from each end of the box to the most extreme values within 1.5 interquartile ranges. Outliers are data beyond the ends of the whiskers. Regression analyses according to the best fitting model were performed to calculate regression lines for the development of total clinical scores over time per group.

For multiple sample comparison of normally distributed data, multiple range test (parametric test) was used to compare means. Kruskal-Wallis test (non-parametric test) was applied to multiple samples with non-normal distribution to compare medians. To compare the medians of two groups, Mann-Whitney-Wilcoxon *W* test was used. In the latter, the lowest achievable probability level was 93% due to small sample sizes (n) between n = 2 and n = 6 per group. Thus, *P*-values below P≤0.07 were accepted as statistically significant. For all tests, *P*-levels are given with the results.

## Supporting Information

Figure S1 **Dose titration and time course of the humoral immune response to** ***C. psittaci*** **infection in calves.** Whole-cell proteins of *C. psittaci* DC15 were separated by SDS-PAGE. Development of the specific antibody response at three different infectious doses in serum (A) and BALF supernatants (B) were analyzed by immunoblotting (no BALF samples from 14 dpi available). Molecular mass markers (kD) are indicated on the right.
(TIF)

Figure S2 **Scheme of intra-bronchial inoculation.**
(TIF)

Table S1 **Clinical Scoring.**
(DOC)
