# Supplementary figures and images for: A Bovine Model of Respiratory Chlamydia psittaci Infection: Challenge Dose Titration
Source: PLoS One. 2012 Jan 27;7(1):e30125. doi: 10.1371/journal.pone.0030125 (PMC3267716; doi:10.1371/journal.pone.0030125)

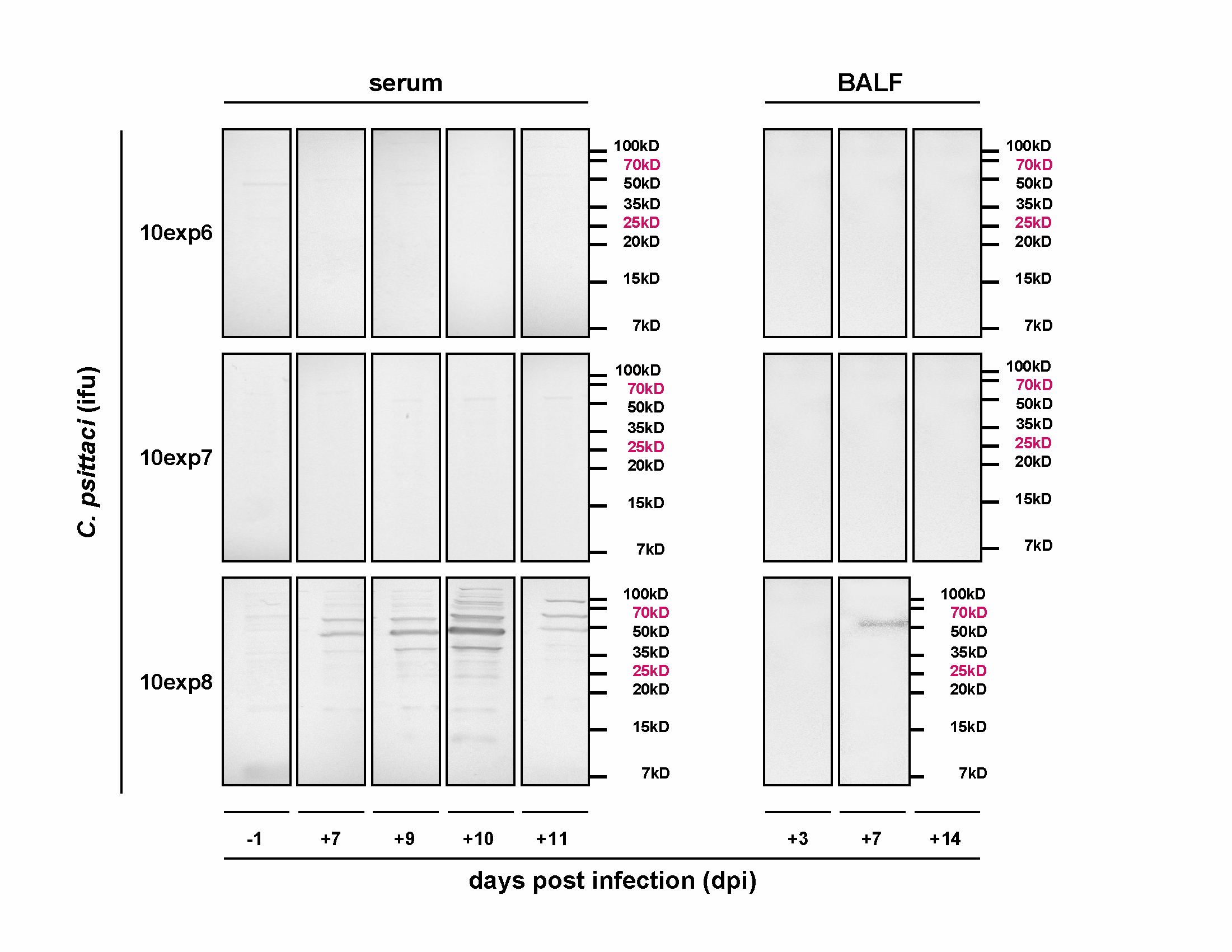

Supplement: Figure S1 — Dose titration and time course of the humoral immune response to C. psittaci infection in calves. Whole-cell proteins of C. psittaci DC15 were separated by SDS-PAGE. Development of the specific antibody response at three different infectious doses in serum (A) and BALF supernatants (B) were analyzed by immunoblotting (no BALF samples from 14 dpi available). Molecular mass markers (kD) are indicated on the right. (TIF) [file pone.0030125.s001.tif]

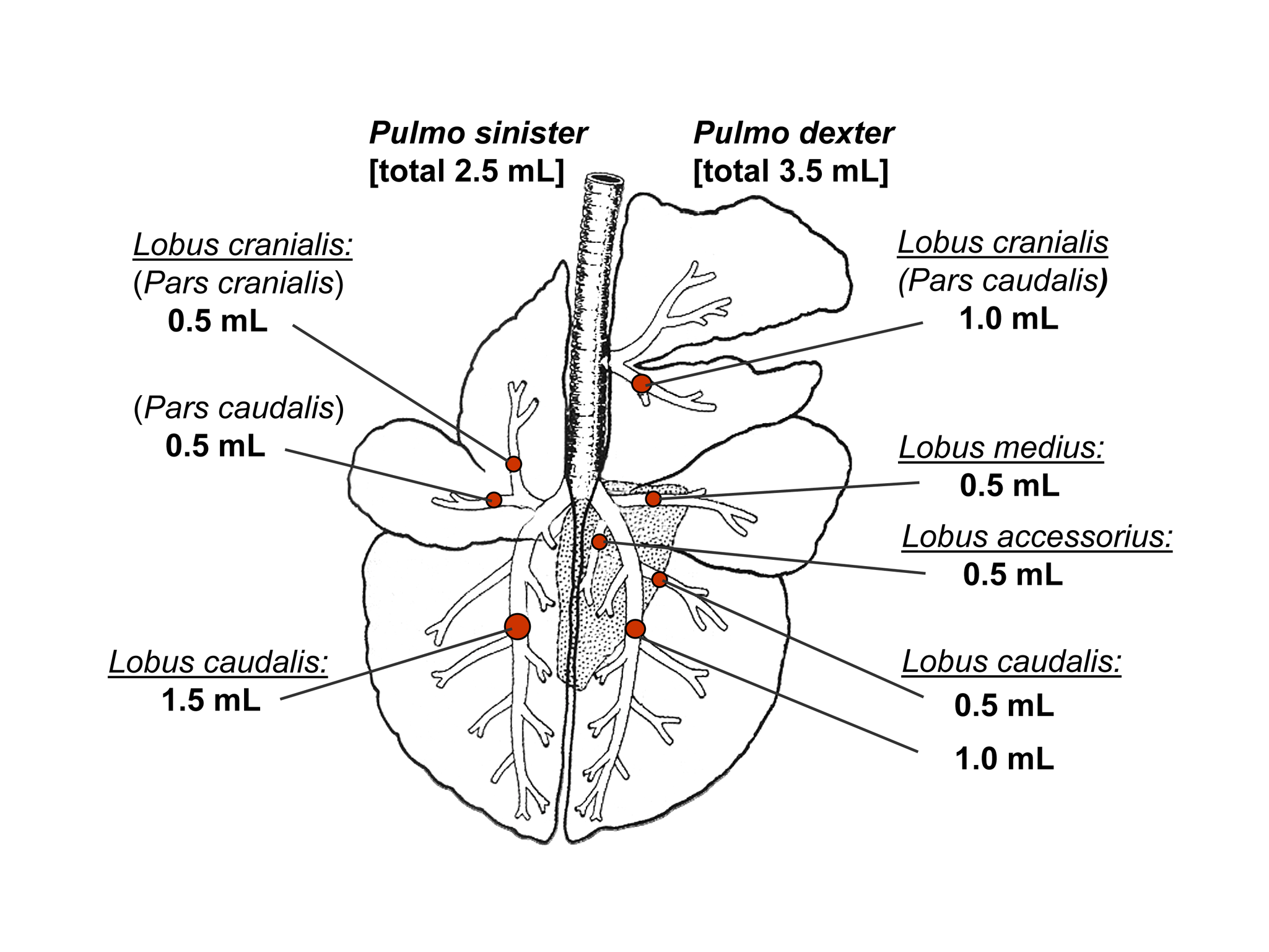

Supplement: Figure S2 — Scheme of intra-bronchial inoculation. (TIF) [file pone.0030125.s002.tif]
