# Supplementary material for: A Bovine Model of Respiratory Chlamydia psittaci Infection: Challenge Dose Titration
Source: PLoS One. 2012 Jan 27;7(1):e30125. doi: 10.1371/journal.pone.0030125 (PMC3267716; doi:10.1371/journal.pone.0030125)
Supplement: Table S1 — Clinical Scoring. (DOC) [file pone.0030125.s003.doc]

**Supplement Table 1:** Clinical Scoring

|  |  | Score [points] | | | | |
| --- | --- | --- | --- | --- | --- | --- |
|  |  | 0 | 1 | 2 | 3 | max. |
| General Condition | **appetite (feed intake)** | **normal** | **reduced** | **no intake** | **-** | 2 |
| (max. 8 points) | **conduct/behaviour** | **lively and vigilant** | **dull: rests a lot** | **very dull: prefers to lie** | **apathetic/moribund** | 3 |
|  | **rectal temperature** | **≤ 39.5 °C** | **39.6 – 40.0 °C** | **40.1 – 40.5 °C** | **≥ 40,6 °C** | 3 |
| Respiratory System | **respiratory rate** | **≤ 30 per min** | **31-40 per min** | **41-50 per min** | **≥ 50 per min** | 3 |
| (max. 17 points) | **nasal discharge** | **none** | **mild/watery** | **moderate/mucous** | **severe/purulent** | 3 |
|  | **ocular discharge** | **none** | **mild/watery** | **moderate/mucous** | **severe/purulent** | 3 |
|  | **spontaneous cough** | **none** | **rarely** | **occasionally** | **frequently** | 3 |
|  | **induced cough** | **none** | **single cough** | **bout of coughing** | **-** | 2 |
|  | **dyspnoea** | **none** | **slight effort** | **moderate effort** | **severe effort** | 3 |
| Cardiovascular System | **heart rate** | **≤ 100 beats/min** | **101-120 beats/min** | **121-140 beats/min** | **≥ 141 beats/min** | 3 |
| (max. 13 points) | **conjunctivae** | **pink, wet, smooth, glossy** | **mild hyperaemia or anaemia** | **mild hyperaemia or anaemia** | **plus 1 point for each yellowish, altered, surface dry** | 5 |
|  | **oral mucosa** | **pink, wet, smooth, glossy** | **mild hyperaemia or anaemia** | **mild hyperaemia or anaemia** | **plus 1 point for each yellowish, altered, surface dry** | 5 |
| Other Organs | **faeces** | **pasty** | **semi solid** | **liquid** | **watery** | 3 |
| (max. 11 points) | **skin and hair** | **skin and hair intact** | **hairless areas** | **decubitus** | **-** | 2 |
|  | **umbilicus** | **not thickened or painful** | **omphalitis** | **-** | **-** | 1 |
|  | **extremities (articulations)** | **normal** | **mono- or oligoarthitis** | **polyarthritis** | **-** | 2 |
|  | ***Lnn. mandibulares* (size)** | **not enlarged** | **low-grade enlarged** | **high-grade enlarged** | **-** | 2 |
|  | ***Lnn. mandibulares* (algesia)** | **not painful** | **painful** | **-** | **-** | 1 |
